# Applying an Indigenous Connectedness Framework to Examine Environmental Risk and Protective Factors for Urban American Indian Children’s Executive Function Development

**DOI:** 10.3390/bs14121202

**Published:** 2024-12-14

**Authors:** Alexis Merculief, Monica Tsethlikai, Felix Muniz

**Affiliations:** 1 Center on Early Childhood, Stanford University, Stanford, CA 94305, USA; 2 Sanford School of Social and Family Dynamics, Arizona State University, Tempe, AZ 85287, USA; monica.tsethlikai@asu.edu; 3 Center for Indigenous Health, Johns Hopkins University, Baltimore, MD 21231, USA; fmuniz1@jh.edu

**Keywords:** American Indian, indigenous, executive function, self-regulation, environment, culture, risk, resilience, connectedness

## Abstract

Indigenous frameworks suggest environmental risk and protective factors for American Indian (AI) children’s development can be understood in terms of connecting and disconnecting forces in five domains: spirituality, family, intergenerational ties, community, and environment/land. This study examined the prevalence of these forces among 156 urban AI parents and their children (mean age = 10.69, *SD* = 1.92) and investigated associations with child executive function (EF). Parents reported on three disconnecting forces (parent stressful life events, discrimination, and neighborhood risks) and two connecting forces (knowledge of tribal history and engagement with cultural beliefs and traditional practices). Parents rated children’s EF using the Behavior Rating Inventory of Executive Function (BRIEF), and a subsample of children (*n* = 81) provided self-report EF data. Controlling for income and child age, connecting forces (parent engagement with cultural beliefs and traditional practices and knowledge of tribal history) were associated with higher parent-reported and child self-reported EF, while disconnecting forces (discrimination and neighborhood risk) were related to lower child EF. Findings highlight the protective role of cultural connectedness for urban AI children’s cognitive development, and the importance of centering Indigenous theory in risk and resilience research with AI families.

## 1. Introduction

Risk and protective factors related to children’s health and wellbeing exist within multilayered and intertwined aspects of the individual, family, society, and the broader environment [1,2,3]. Existing research on risk factors for American Indian and Alaska Native (AI/AN) or Indigenous families historically emphasized greater risk at the individual (e.g., depression, suicide, and substance use disorders) [4] and family or household levels (e.g., low socioeconomic status or adverse childhood experiences) [5,6]. This study is one of the first to explore the influence of environmental risk factors, alongside protective factors such as cultural connectedness, on developmental outcomes for urban AI/AN children. Strong executive functioning (EF) represents one key developmental outcome in childhood [7,8], as well as a protective factor for wellbeing into adulthood [9,10]. EF skills (e.g., working memory, inhibition, and cognitive flexibility) [11] help children navigate and manage their emotions and behavior within the demands of their community context, and predict academic achievement [12], positive peer relationships [13], better mental health [14,15], and lower substance use [16]. While environmental and socioeconomic risks have been associated with lower EF in the general population [17,18], little research explored the impact of environmental risks on EF for urban AI/AN children. Furthermore, recent government mandates increasingly echo Indigenous leaders in encouraging the use of Indigenous ways of knowing in scientific research [19]. Perspectives such as the Indigenous connectedness framework [20] move beyond individual and socioeconomic factors and reconceptualize protective factors as connecting forces and risk factors as disconnecting forces [20,21]. Under this framework, children’s environments consist of five connectedness mechanisms (referred to in this paper as “domains”) that holistically influence child wellbeing and thriving: spirituality, family, intergenerational ties, community, and environment/land [20]. Thus, the present study applies the Indigenous connectedness framework to examine environmental risk and resilience as connecting and disconnecting forces for urban American Indian (AI) children and families in relation to children’s developing executive function skills.

### 1.1. Child Executive Function Skills (EF)

EF is defined as a set of separable but related cognitive skills that allow individuals to process multiple sources of information at once (working memory), inhibit pre-potent thoughts and responses (inhibitory control), and shift flexibly between tasks (cognitive flexibility) [22,23,24]. EF skills allow children to align their behavior and emotions with their social contexts, which for AI/AN children may include a classroom, listening to stories told by a community elder, playing sports, attending spiritual ceremonies or religious services, or dancing at a Pow Wow. As children grow older, these skills become more volitional (e.g., intentional self-regulation) [25] and they use these skills to align their behavior with their emerging identity (including their cultural identity) and who they want to be in the world. Culturally grounded conceptualizations of EF have been discussed (i.e., emphasizing balance between one’s emotions, body, mind, and spirit) [26], but measures have not yet been designed. Some well-established direct assessments of EF (e.g., backward digit span, the Wisconsin Card Sorting Task) have been utilized with Indigenous children, but questions remain about their cultural validity [27]. More nuance may be gleaned using subjective measures (e.g., the Behavior Rating Inventory of Executive Function or BRIEF) [28,29] which assess child behavior using real-world examples of EF (e.g., “needs help to stay on task”, “when given a list of things to do, only remembers the first”). While some aspects of these measures may still lack cultural relevance, subjective measures can incorporate cultural context by centering the rater’s perspective (AI/AN parents or children themselves), and they remain some of the only tools available to measure EF while the field awaits culturally grounded assessments. Indeed, prior research utilizing parental reports on the BRIEF found that higher levels of cortisol (a proxy indicator of stress) in urban AI children’s hair was related to poorer working memory and cognitive flexibility [30]. Importantly, EF and related skills are developed in the context of rich and supportive social relationships and are impacted by risk and protective factors in both the child’s and parent’s environments [11,31]. Thus, the present study examines the influence of connecting and disconnecting forces in urban AI children’s environments in relation to EF behavioral ratings, as measured by both parent and child reports.

### 1.2. Connecting and Disconnecting Forces and Children’s Executive Function

#### 1.2.1. Environmental Disconnectedness

Children’s environments are composed of physical (or “built”) and social aspects, which share reciprocal influence on development [32,33]. Examples of risks in the built environment include lack of access to green spaces, housing, health care, and food, as well as poor water and air quality [33,34]. Risks in the social environment encompass residential safety and crime, hygiene, presence of drugs and alcohol, and gangs [35,36]. For AI/AN children, the environment may also include a deep connection to one’s tribal lands [20,37], expressed through caring for plants and animals, gathering traditional foods [20], and protecting the land, which have been continually exploited by settler colonialism, industrialization, and ongoing toxic exposures [38]. Only a handful of published studies explored environmental risks, particularly neighborhood risks, in AI/AN communities, finding that AI/AN families living on tribal reservations are exposed to higher crime [39,40] and greater access to drugs and other substances [41]. While neighborhood risks are associated with poor cognitive outcomes for children in the general population and with other minoritized communities [42,43,44], previous studies with AI/AN families living on tribal reservations found no relation between neighborhood risk and children’s EF [45].

To unpack these differences, frameworks such as the phenomenological variant of ecological systems theory [46] emphasize the importance of considering individuals’ perceptions of their environments, not just objective characteristics of the environment itself. As stated by Beale Spencer “Social science has always benefited from analyses that provide perspectives of others outside the person. However, we restate that a systems-oriented analysis that acknowledges the critical and ever-present role of the person’s own phenomenology or unique set of perceptions is also needed” [46]. For example, health impacts differ based on the subjective appraisal of risks and the level of perceived stress associated with them [47,48,49]. Indeed, despite the well documented high poverty and greater access to drugs and alcohol, AI/AN parents do not perceive their neighborhoods as risky using typical metrics [45]. Furthermore, a combination of political and socioeconomic factors contributed to many AI/AN families moving off tribal lands and into cities (upwards of 70%) [50]. These include forced relocation [40,51], the removal of AI/AN children from their families during the boarding school era [52], and various government incentives for AI/AN families to move to urban areas (e.g., the Indian Relocation Act of 1957) [53]. However, the experiences of urban AI/AN families remain understudied. The present study thus advances environmental health equity for urban AI families by examining perceptions of neighborhood risk as one aspect of environmental disconnectedness and investigates associations with child EF.

#### 1.2.2. Family Disconnectedness

The domain of the family or home can bring features of support and connectedness for AI/AN children, as well as risk factors. These may include socioeconomic factors, such as a parent or caregiver’s education, employment, and income. Low socioeconomic status (SES) has long been associated with negative developmental outcomes, including lower academic achievement [54] and poorer EF in the general population [55,56,57]. However, some studies with AI/AN communities failed to find evidence of an association between SES and EF for AI children living in reservation communities [30,45,58]. Beyond SES, other risk factors related to the home or family include stressful life experiences such as abuse and neglect, and living with a family member who struggles with mental health, suicidality, or substance use [59]. AI/AN children are disproportionately exposed to these adverse experiences [60], and while evidence is limited in AI/AN communities, these experiences are consistently related to poorer child EF in the general population [61]. Within the Indigenous Connectedness Framework, family connectedness can be understood as fulfilling cultural and relational roles and responsibilities and maintaining feelings of togetherness, trust, and safety [20]. Disconnectedness may then occur when that trust and safety is broken, as it is when children and families undergo adverse and stressful life experiences such as abuse and neglect. Therefore, the present study examines parent/caregiver reports of stressful life events (a measure of adverse experiences) [62] as one aspect of family disconnectedness and investigates associations with urban AI child EF.

#### 1.2.3. Community Disconnectedness

A sense of belonging in one’s community is vital for every child’s wellbeing [48] and is especially protective for children from minoritized communities [63,64,65]. Community connectedness for AI/AN families might also appear as cooperation and sharing of resources, participating in traditions and ceremonies, engaging in collective governance, and simply living, working, and celebrating with one’s community [20]. However, for urban AI/AN families, living and engaging with the community of the city or town they live in may also bring experiences of racism, prejudice, and discrimination [66]. AI/AN families living in cities that border tribal reservations may experience this to a greater extent, as the residents from the dominant cultural group may have closer historical and political relations with the nearby tribe, and greater access to stereotypes about American Indian or Indigenous peoples [66]. Lack of community belonging, prejudice, and discrimination are related to poor child mental health and lower academic achievement for adolescents and young adults in both AI/AN reservation communities and other minoritized communities [67,68,69], with some evidence of negative impacts on EF and related skills [70,71]. Parent’s experiences of racial discrimination can also manifest as vicarious racism and have been shown to impact their children’s mental health and cognitive development [72]. However, little is known about urban AI/AN experiences of discrimination or impacts on EF in early childhood. Therefore, the present study examines parent/caregiver reports of discrimination as one aspect of community disconnectedness and investigates associations with AI child EF.

#### 1.2.4. Intergenerational Connectedness

While Western literature is lacking with regard to research on the importance of intergenerational connectedness, the Indigenous connectedness framework describes this level of connection as a knowledge and awareness that one is part of a continuous history and ancestral line [20]. An appropriate knowledge of one’s tribal history, ancestors, and the historical trauma that ancestors endured and moreover persevered to maintain and pass on culture leads Indigenous people to an awareness that they are both a part of a strong, unbroken ancestral line and they are responsible for passing this on to future generations. While the negative impacts of intergenerational and historical trauma on the health and wellbeing of Indigenous children are established [73], intergenerational connectedness acts as a protective factor and is associated with better mental health [74], socio-emotional skills [75,76], and a more secure identity [74,77] for both Indigenous and non-Indigenous children and youth. For Indigenous people, intergenerational connectedness may include engaging in intergenerational narratives and storytelling, living in multigenerational households, participating in one’s tribal governance, learning tribal history from elders, and learning one’s language. Research linking these factors to AI/AN children’s EF skills in preschool is sparse, particularly in urban AI/AN contexts. The present study is one of the first to examine associations between parents’ intergenerational connectedness (as measured by knowledge and reported impact of their tribal history) and AI child EF.

#### 1.2.5. Spiritual Connectedness

Indigenous elders, parents, and community members routinely place the importance of children’s spiritual and cultural identity and connectedness above most other childhood outcomes [78]. Spiritual connectedness is related to intergenerational connectedness, with shared aspects including speaking and hearing one’s tribal language and maintaining a connection with one’s ancestors [20]. However, spiritual and cultural connectedness (intertwined and often interchangeable constructs) also include participating in tribal ceremonies such as naming or puberty ceremonies, eating traditional foods, engaging in prayer, using traditional medicine, participating in art and dance, and maintaining a connection with community, nature, and all living things [20,79,80]. Strong cultural identity and spiritual connectedness may be related to better mental and behavioral health [39,79,81,82,83] and academic achievement [84,85,86] for AI/AN children and youth. Indeed, AI/AN parents’ own cultural identity and engagement in traditional practices can positively impact children’s cultural identity and socioemotional development [87,88], especially as Indigenous children readily learn by observation [89]. While literature is sparse connecting AI/AN culture and spirituality to EF outcomes, some studies find that parents’ cultural socialization practices are associated with better EF for AI/AN children. For example, one study found that parents’ use of tribal language socialization strategies, participation in tribal ceremonies, and playing AI/AN games with their children were associated with higher EF for AI/AN preschool children living on tribal reservations [45]. Another study found that AI children with some tribal language knowledge had better inhibitory control and working memory skills than children with no tribal language knowledge [90]. However, maintaining spiritual and cultural connectedness can be more difficult for urban AI/AN families, as they are isolated from their tribal communities. Providing their children with access to cultural activities and spiritual guidance may require considerable time and financial resources from urban AI/AN parents, including driving long distances to urban cultural centers or nearby tribal reservations. Little is known about the relation between spiritual or cultural connectedness and child cognitive outcomes for urban AI/AN communities. Thus, the present study examines parent/caregiver’s cultural beliefs and traditional practices as one aspect of spiritual connectedness and investigates associations with AI child EF.

### 1.3. Present Study

Three main research aims guided the present study. Due to the novelty of this research with urban AI/AN families, these aims were primarily exploratory in nature. The first aim examined the prevalence and co-occurrence of risk and protective factors through the lens of disconnecting and connecting forces for urban American Indian (AI) families with children ages 7–14 years old. Parent-reported connecting forces were spiritual connectedness (as measured by cultural beliefs and traditional practices) and intergenerational connectedness (measured by knowledge of tribal history), while disconnecting forces were environmental disconnectedness (measured by neighborhood risk), community disconnectedness (measured by discrimination), and family disconnectedness (measured by stressful life events). The second aim investigated whether connecting and disconnecting forces were related to parent-rated child executive function as measured by the BRIEF [28]. Given null findings from prior literature with tribal populations [45], we anticipated that environmental disconnectedness (i.e., neighborhood risk) would not be related to child EF, but other aspects of disconnectedness (i.e., stressful life events and discrimination) would be related to lower child EF. We also anticipated that connecting forces (i.e., parents’ cultural beliefs and traditional practices and knowledge of tribal history) would be related to higher child EF. The third aim utilized a subsample of older children (ages 11–14) with self-report data to examine these same connecting and disconnecting forces as predictors of child-self reported EF. By including child self-reports alongside parent reports, this aim sought to explore potential discrepancies between how parents and children perceive the child’s EF and provide novel insights into how connecting and disconnecting forces in the environment differentially influence AI children’s and parents’ perceptions of EF.

## 2. Materials and Methods

### 2.1. Study Design

Urban American Indian (AI) parents and caregivers and their children were recruited from a school district in the Southwest U.S. A long-term collaborative relationship with the directors of the Native American Education Program facilitated data collection from 2015 to 2017, with the parent advisory board approving all questionnaires and study procedures. The study was approved by both the participating school district and the institutional review board (IRB) of the primary investigator’s (second author) university. The university’s IRB conducts an additional review of Indigenous research to ensure proper cultural protocols are followed in addition to the main IRB review. Tribal IRB approval was not solicited as all participants resided in urban areas. In two cases where participants were found to live on reservation lands, the participants were informed they could not participate due to not having the consent of their tribal government. All participants (regardless of eligibility) received USD 50 for the primary caregiver (as well as USD 50 for the participating children) for meeting with the research team. Parents consented and children assented to the study procedures, related surveys, and assessments.

### 2.2. Participants

Participants included 156 urban AI parents and caregivers with children between the ages of seven and fourteen years old (mean age = 10.69, *SD* = 1.92; 57.05% female). The third aim of the study utilized a subsample of 81 older children ages 11 to 14 (mean age = 12.27, *SD* = 1.06) with self-report EF data. Most of the respondents to the parent/caregiver survey (84.62%) identified as the child’s biological or adoptive mother or father, with other caregivers identifying as a grandparent or other relative. Caregivers will be referred to as “parents” in the present study. All children were identified by their parent as American Indian, either alone or with another racial/ethnic identity. To protect the participants, information on specific tribal affiliation is not available. Average annual household income was reported as USD 43,197.01 (SD = USD 31,182.81), with 38.89% of families at or below the federal poverty level for their household size [91].

### 2.3. Measures

Parents were asked to complete a set of four questionnaires plus demographic questions administered electronically via cell phone, computer, or tablet through Qualtrics. The primary investigator and/or research team members were available to assist parents with the questionnaires as needed.

#### 2.3.1. Neighborhood Risks (Environmental Disconnectedness)

Core survey items originated from the “Neighborhood Problems” and “Environmental Conditions” scales embedded in the Head Start AI/AN Family and Community Experiences Study, a large national dataset encompassing primarily tribal reservation communities [92,93]. These items asked participants to rate various neighborhood concerns using a three-point Likert scale where 1  =  “not a problem”, 2  =  “somewhat of a problem”, and 3  =  “big problem”. Items encompassed aspects of risk in the built environment (e.g., “not enough housing”, “not enough jobs”) and social environment (e.g., “crime”, “violence”, “unsupervised children”). Due to the small number of responses for the “big problem” category, responses to all items were binary coded such that 0 = risk absent (“not a problem”) and 1 = risk present (“somewhat” or “big” problem). After analyzing interitem correlations and scale reliability, some items were dropped due to lack of variability, high missingness, or conceptual overlap with other items in the subscale (correlation greater than *r* = 0.70). The final neighborhood risk scale contained 15 items (Appendix A, Table A1), with a scale reliability of α = 0.92. For hypothesis testing, the total number of risks indicated by parents was calculated for participants with at least 80% complete data.

#### 2.3.2. Stressful Life Events (Family Disconnectedness)

Parents were presented with a list of nine stressful life events (Appendix A, Table A1) and asked to report if they experienced them in the past year (yes/no), such as “Has someone important to you committed suicide?” or “Have you been physically abused or hurt by a spouse, boyfriend, or girlfriend?” The items included in the stressful life events questionnaire were adapted from previous questionnaires and have been used in prior research in AI communities [62]. Scale reliability for the stressful life events scale was α = 0.71. Similar to the calculation for neighborhood risks, affirmative answers were summed to calculate the total number of stressful life events experienced for participants with at least 80% complete data.

#### 2.3.3. Perceived Discrimination (Community Disconnectedness)

Perceptions of discrimination were measured using items adapted from the everyday discrimination scale [94]. Parents were presented with a list of four statements related to discrimination (Appendix A, Table A1), for example: “You have problems in stores or restaurants because you are American Indian”. Participants were asked to rate these on a three-point Likert scale (“not a problem”, “some problems”, “a lot of problems”). The scale reliability for the discrimination scale was α = 0.82. Similar to the calculation for neighborhood risks, responses to all items were binary coded, where 0 = risk absent (“not a problem”) and 1 = risk present (“some” or “a lot” of problems), such that higher scores indicate more perceptions of discriminatory experiences. For hypothesis testing, the total number of discrimination items experienced was calculated for participants with at least 80% complete data.

#### 2.3.4. Cultural Beliefs and Traditional Practices (Spiritual Connectedness)

Parents were presented with a list of eight items (Appendix A, Table A2) that measure cultural identity, beliefs, and engagement with traditional and spiritual practices adapted from items used in prior research in AI communities [95,96]. Examples include: “I speak or am learning to speak my tribal or cultural language” and “I have a strong sense of belonging to my own tribe”. Participants were asked to rate the level of agreement with these items on a five-point Likert scale (strongly disagree—strongly agree), with higher scores indicating greater importance of cultural beliefs and engagement with traditional practices. The scale reliability for the cultural beliefs and traditional practices scale was α = 0.81. For hypothesis testing, average cultural beliefs/traditional practices scores were calculated for participants with at least 80% complete data.

#### 2.3.5. Knowledge and Impact of Tribal History (Intergenerational Connectedness)

The items assessing parental knowledge and the perceived impact of tribal history were adapted from a measure of historical consciousness developed by The American Indian Service Utilization, Psychiatric Epidemiology, Risk and Protective Factors project [97]. Parents were presented with a list of four statements (Appendix A, Table A2), for example: “How much do you know about your tribal history- over the past 150 years or so? “and “How big of an impact has tribal history had on your life?” Participants were asked to rate the impact of each item on a three-point Likert scale (“not at all”, “somewhat/some impact”, or “a lot/a lot of impact”), with higher scores indicating greater knowledge and impact of tribal history. The scale reliability for the tribal history scale was α = 0.72. For hypothesis testing, average tribal history scores were calculated for participants with at least 80% complete data.

#### 2.3.6. Child Executive Function

Child executive function was measured via the Behavior Rating Inventory of Executive Function parent report [28], and for the third aim of the study, child self-report forms designed for children 11 through 18 years old [29]. Both parents and children (Appendix A, Table A3) were presented with various statements that reflect aspects of executive function and asked to rate them on a three-point Likert scale (“never”, “sometimes”, or “often”). From the parent report form, three subscales were utilized: the working memory subscale (10 items, “Forgets what she/he was doing”; α = 0.90), the inhibitory control subscale (10 items, “Interrupts others”; α = 0.89), and shifting subscale (henceforth referred to as “cognitive flexibility”; eight items, “Resists change of routine, foods, places”; α = 0.88). The child self-report form also utilized the working memory subscale (12 items, “I forget what I am doing in the middle of things”; α = 0.87), inhibitory control subscale (12 items, “I have trouble sitting still”; α = 0.87), and cognitive flexibility subscale (10 items, “I get stuck on one topic or activity”; α = 0.78). Items were reverse coded such that higher scores = better EF. For hypothesis testing, scale averages were created for participants with at least 80% complete data.

#### 2.3.7. Covariates

Child age, sex, and socioeconomic status are frequently related to executive functioning in the literature [11], but child sex was not correlated with any predictor or EF outcome and was trimmed from all models. Child age was included as a covariate in models using the full sample due to the wider age range of children (ages 7–14 years). However, in order to preserve power, it was trimmed from exploratory analyses utilizing the smaller subsample of older children due to a narrower age range (ages 11–14), and because it was not related to any predictor or outcome in those models.

### 2.4. Analytic Strategy

For analyses using the full sample, missingness on parent-rated constructs (connecting and disconnecting forces and parent-reported child EF) ranged from 8.97% to 13.89%. For analyses using the subsample of older children, missingness on parent-rated constructs was similar, but missingness for child self-reported EF via the BRIEF was 30.0%. Missingness on parent-reported constructs was not related to any demographic characteristics, while children missing the BRIEF survey measures were younger (*M* = 11.77, *SD* = 1.11; *n* = 81) than children with complete data (*M* = 12.64, *SD* = 1.09; *t*(82) = 3.37, *p* = 0.001 (per developer guidelines, the BRIEF is only appropriate for use with children ages 11 and older and with a 5th grade reading level, and some of the younger children in this age window were not able to complete the self-report)). For hypothesis testing, missingness was handled using full information maximum likelihood (FIML) in all models.

All statistical analyses were run using STATA version 18.0 software [98]. To answer the first research question, descriptive statistics investigated the prevalence of connecting and disconnecting forces as reported by urban AI parents. Co-occurrence and relations between these connecting and disconnecting forces were investigated using pairwise spearman correlations. To answer the second research question, which investigated the association between connecting and disconnecting forces and parent-rated child EF, linear regressions were run in an SEM framework, controlling for family income and child age. Connecting and disconnecting forces were tested as concurrent predictors, with separate models for each BRIEF subscale (working memory, inhibition, and cognitive flexibility). Multicollinearity was checked and variance inflation factor (VIF) values were below 2.0 for all predictors in all models, well below the threshold of 10.0 indicating multicollinearity concerns [99]. The third (exploratory) aim utilized a similar statistical strategy with the subsample of older children with self-report data (ages 11–14) and investigated connecting and disconnecting forces as concurrent predictors of each BRIEF subscale, comparing parent and child self-reported EF, and controlling for family income.

## 3. Results

### 3.1. Prevalence and Co-Occurrence of Connecting and Disconnecting Forces

Parents reported low to moderate levels of environmental disconnectedness (Appendix A, Table A1), as measured by neighborhood risk. On average, parents reported the presence of 4.43 out of fifteen neighborhood risks (*SD* = 4.65). Close to 20% of the sample did not report any of the stated risks as problematic in their neighborhood or community. The most common risks reported were “not enough jobs” (46.72%), “unsupervised children” (45.71%), and “high theft” (39.44%). Similarly, parents reported low levels of family disconnectedness as measured by stressful life events. Parents reported experiencing an average of 1.49 (*SD* = 1.84, min = 0.0, and max = 7.0) stressful life events in the past year. The most common stressful events included “violence between members of your family” (38.97%) and “physical abuse (non-intimate partner)” (19.85%). Parents reported experiencing 1.19 out of the four possible discrimination items (*SD* = 1.41), indicating low to moderate community disconnectedness. The most common response was experiencing prejudice from White members of their community, which nearly half of AI parents reported experiencing to some degree (47.24%).

Conversely, AI parents reported moderate to high levels of intergenerational connectedness, as measured by knowledge and impact of their tribal history (Appendix A, Table A2). The average score was 1.99 (*SD* = 0.61, min = 1.0, and max = 3.0), which was closest to “some/somewhat” on the Likert scale used to measure these items. Last, parents reported moderate to high spiritual connectedness (Appendix A, Table A2), as measured by engagement with cultural beliefs and traditional practices. The average agreement with these beliefs and practices was 3.69 (*SD* = 0.81, min = 1.0, and max = 5.0), which corresponded with “somewhat agree” on the Likert scale used to measure these items. Some of the most common beliefs and practices were “Being a part of my tribe is important to me” (51.56% strongly agreed), “I have a lot of pride in my tribe or cultural group”, (49.22% strongly agreed), and “I speak or am learning to speak my tribal language” (30.47% strongly agreed).

Spearman correlations (Table 1) tested the associations between connecting and disconnecting forces. Parents who reported the presence of more environmental disconnectedness (neighborhood risks) were more likely to also report experiencing more family disconnectedness (stressful life events; rho = 0.39, *p* < 0.001) and more community disconnectedness (discrimination; rho = 0.32, *p* < 0.001). The two connecting forces (engagement with cultural beliefs and traditional practices and knowledge and impact of tribal history) were moderately correlated with each other (rho = 0.38, *p* < 0.001). One disconnecting force, discrimination, was related to higher cultural engagement (rho = 0.28, *p* = 0.01). However, neither environmental nor family disconnectedness (neighborhood risk or stressful life events) were related to connecting forces for parents. Critically, family income was not significantly related to any connecting or disconnecting forces for urban AI families.

### 3.2. Connecting and Disconnecting Forces and Parent-Rated Child EF

Parents rated their children’s EF using the BRIEF working memory, inhibition, and cognitive flexibility subscales (Appendix A, Table A3). Linear regressions investigated connecting and disconnecting forces as concurrent predictors of parent-reported child EF (Table 2), controlling for family income and child age. As expected, higher community disconnectedness, as measured by parents’ experiences of discrimination, related to lower inhibition (*β* = −0.23, SE(*β*) = 0.09, *p* = 0.01), working memory (*β* = −0.22, SE(*β*) = 0.10, and *p* = 0.02), and cognitive flexibility (*β* = −0.33, SE(*β*) = 0.09, and *p* < 0.001).

**Figure 1:**
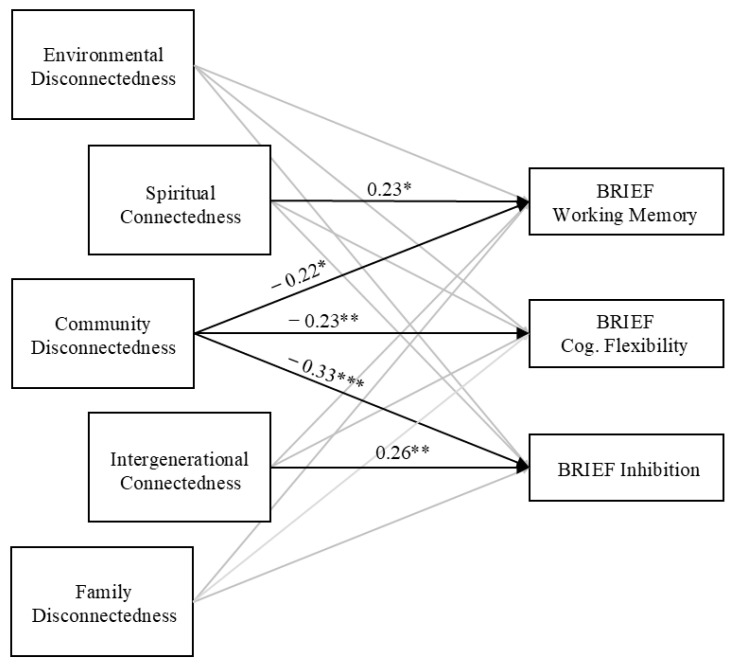
Connecting and disconnecting forces and parent-reported child EF (*n* = 156). Identical independent models run with each parent-reported BRIEF subscale as outcome. All models controlled for family income and child age. Standardized betas reported, nonsignificant paths not shown. * *p* < 0.05, ** *p* < 0.01, and *** *p* < 0.001.

Additionally in alignment with our hypotheses, connecting forces were related to higher child EF (Figure 1). Spiritual connectedness, as measured by parents’ cultural beliefs and traditional practices, was associated with higher working memory (*β* = 0.23, SE(*β*) = 0.11, and *p* = 0.02), while intergenerational connectedness, as measured by parents’ knowledge of tribal history, was related to higher inhibition (*β* = 0.26, SE(*β*) = 0.10, and *p* = 0.005). Neither environmental disconnectedness (as measured by neighborhood risk) nor family disconnectedness (as measured by stressful life events) were related to child EF. The null findings for environmental disconnectedness were in line with our hypotheses, but the null findings for family disconnectedness were not expected, as we had anticipated that parent stressful life events would be related to lower child EF.

### 3.3. Connecting and Disconnecting Forces and Child Self-Rated EF

A subsample of children ages 11–14 (*n* = 81) and their parents rated children’s EF using the BRIEF working memory, inhibition, and cognitive flexibility subscales (Appendix A, Table A3). Paired t-tests indicated that parents and children reported similar mean levels of working memory and inhibition, but parents reported higher cognitive flexibility (*M* = 2.64, *SD* = 0.35) than children (*M* = 2.46, *SD* = 0.34; *t*(47) = 2.23, and *p* = 0.03). However, child self-report and parent-reported subscales were not significantly correlated with one another (Table 1), indicating inconsistent agreement between parents and children regarding children’s EF. Linear regressions investigated the relation between family connecting and disconnecting forces and child self-rated EF, compared with parent ratings of child EF for this subsample. Controlling for family income, relations between connecting and disconnecting forces and parent ratings of older children’s EF (Table 3) were generally in alignment with the results for the full sample, although intergenerational connectedness was no longer a significant predictor of parent-rated EF for this subsample.

Models using child self-reported EF (Table 3) mirrored findings for parent-rated EF in one key way. Spiritual connectedness, as measured by parent engagement with cultural beliefs and traditional practices, was related to higher child self-rated EF (inhibition subscale; *β* = 0.45, SE(*β*) = 0.13, and *p* = 0.001). In alignment with the findings for parent-rated EF, neither family disconnectedness nor intergenerational connectedness were related to child self-rated EF. Child self-report data differed from parent ratings in that community disconnectedness (as measured by parent experiences of discrimination) was not significantly related to child self-reported EF. Interestingly, greater environmental disconnectedness, as measured by neighborhood risk, predicted lower child self-reported cognitive flexibility (*β* = −0.39, SE(*β*) = 0.15, and *p* = 0.01), contrary to our hypothesis. Models with this subsample were underpowered and should be interpreted with caution; however, they provide valuable insights into AI children’s own perspectives on their executive function skills and help address potential biases that may arise from relying solely on parent-reported data.

## 4. Discussion

The present study applied an Indigenous connectedness framework to examine environmental risk and protective factors as connecting and disconnecting forces for urban American Indian (AI) children and families. We also investigated the association between connecting and disconnecting forces in children’s environments and executive function (EF) skills as measured by both parent and child self-report. As anticipated, spiritual and intergenerational connectedness (as measured by engagement with cultural beliefs and traditional practices and knowledge of tribal history) were both associated with higher parent-reported EF, and spiritual connectedness was associated with higher child self-reported EF. While family disconnectedness (as measured by stressful life events) was not related to parent or child self-reported EF, community disconnectedness (as measured by discrimination) was associated with lower parent-reported EF, and environmental disconnectedness (as measured by neighborhood risk) was associated with lower child self-reported EF. These findings provide novel evidence on the prevalence of risk and protective factors present in the environment for urban AI families and emphasize connecting forces as contributors to cognitive health and wellness for AI children. Findings also highlight the relative importance of community disconnectedness as potentially detrimental for urban AI child cognitive health compared with null findings for typical risk factors such as low socioeconomic status.

### 4.1. Connecting and Disconnecting Forces as Environmental Risk and Resilience Factors

Despite living away from their tribal communities, urban AI families reported relatively high spiritual connectedness (as measured by engagement with cultural beliefs and traditional practices) and intergenerational connectedness (as measured by knowledge and impact of tribal history). Over half of parents stated they have a strong sense of belonging to their tribe, and most parents expressed high cultural pride (78.9%). Fewer families participated in cultural activities, such as singing and dancing, or followed traditional religious or spiritual beliefs. This may be a matter of personal choice, but it could also be due to lack of financial or other resources needed to drive to community centers or travel to their tribe to participate in these activities. As for intergenerational connectedness, most AI parents (82.1%) reported they were “somewhat” or “very” familiar with their tribe’s history over the past 150 years. Still, only 16.4% were able to say they were “very familiar”, which is likely due to the isolating nature of living apart from cultural wisdom, elders, and traditional tribal lands. Overall, the present study affirms that urban AI families express a strong desire to connect to their culture. Unfortunately, higher spiritual connectedness was related to higher discrimination, a finding which has been affirmed by other studies [100]. Nearly half of parents reported experiencing prejudice from White members of their community, and 30% reported experiencing problems in stores or restaurants because they were American Indian. The relation between cultural or spiritual connectedness and discrimination is likely because a greater connection to one’s culture may result in “standing out” as more visibly Native, thus opening up urban AI individuals to greater experiences of discrimination in cities where they are the minority.

Urban AI families reported low to moderate levels of other disconnecting forces (neighborhood risk and stressful life events). Close to 20% of families did not report any risks in their neighborhood, and 46% reported zero stressful life events in the past year (e.g., trauma, family violence). Simultaneously, nearly 40% of families were below the federal poverty level, indicating the presence of socioeconomic risk. Despite this, income was not related to any disconnecting forces (discrimination, neighborhood risk, or stressful life events). One explanation is that as an objective metric, annual income alone does not provide information on the relative importance of socioeconomic status for AI families. Lack of material resources may not be perceived as being as great of a stressor as social or community-level stressors, emphasizing the need to focus on socially or culturally disconnecting forces rather than relying on objective measures of risk for urban AI families. Conversely, the subjectivity of items used to measure neighborhood risk and discrimination in the present study allowed AI families to express the relative impact of stressors in their lives. Results suggest future research with urban AI populations may wish to focus on employment and housing availability, street safety (car accidents), crime (theft), and child care resources (to address unsupervised children), as these were the most commonly reported disconnecting forces in the neighborhood environment. Nevertheless, urban AI families rarely described the level of these risks as “big problems” in their community. While this is encouraging, this, along with the floor effects found for other disconnecting forces, indicates that current understandings of environmental risk are missing key elements that AI families experience as particularly problematic or risky in their daily lives. While we await culturally relevant measures of risk and disconnectedness, the present study helps clarify which aspects of environmental risk available in the general literature are more or less relevant for urban AI families.

### 4.2. Connecting Forces and Urban AI Children’s EF

Spiritual and intergenerational connectedness (as measured by engagement with cultural beliefs and traditional practices and knowledge of tribal history, respectively) were related to higher parent-rated executive function for the full sample. These novel findings with urban AI families echo previous studies which demonstrate the positive impacts of cultural connectedness on AI child cognitive development for those living in tribal reservation communities [45,90]. There are many mechanisms through which parent-reported cultural and spiritual connectedness can influence children’s EF. First, parents who are themselves invested in activities such as learning their tribal language and tribal history may be more likely to enroll their children in similar cultural activities, such as participating in AI community camps and sports. Cultural activities provide many rich opportunities for cognitive growth. For example, Indigenous children often learn by observation, requiring attention and focus (skills related to EF) [89]. Indigenous sports are collective and team-oriented, requiring children to inhibit individual goals for that of the team [101], and Pow-Wow dancing, arts, and crafts also require holding many steps and rules in mind (i.e., working memory) while maintaining focus. Even if parents do not enroll their children in child-specific cultural activities, AI children likely attend adult community events, ceremonies, and meetings with their parents, as Indigenous gatherings are not so starkly age segregated as events typical in American dominant culture. This level of access to Indigenous elders, mentors, and peers cannot be understated, as urban AI children are often one of a small percentage of their school’s population of Indigenous children. This access to supportive relationships with adults and peers provides the social foundations for healthy EF development, and spiritual and cultural connectedness also foster healthy identity development for AI/AN youth.

That said, it is also possible that the association between parent-rated spiritual and intergenerational connectedness and child EF was due to AI parents simply perceiving their children’s EF more positively due to the increased mental health, happiness, and balance they achieve because of greater cultural and spiritual connection [79]. Parents with better mental health are more available to provide warm and supportive caregiving for their children, which in turn supports emerging EF [102,103]. To address possible bias embedded in parent ratings of EF, underpowered exploratory analyses also measured associations between connecting and disconnecting forces and child self-rated EF. While parents and children rated the children’s EF at similar mean levels (Appendix A, Table A3), parent and child ratings were not correlated with one another (Table 1). This may be because children’s perceptions of their own self-regulatory abilities frequently include contexts that the parents do not have access to (namely, their schools). Children may be rating their EF and other behaviors in terms of how they see themselves in relation to the demands of their schools. Nevertheless, for models examining child self-rated EF, results continue to show positive relations between greater parent spiritual connectedness and higher child self-rated EF. This lends further evidence to suggest that urban AI parents’ level of cultural engagement has positive effects on the health and wellbeing of the whole family.

Last, it is important to note that EF skills are closely related, and even direct assessments of EF cannot fully tease apart working memory, inhibition, or cognitive flexibility as separable components. Thus, it is difficult to draw conclusions about why spiritual and intergenerational connectedness were more related to working memory and inhibition, while we did not find significant associations with cognitive flexibility. It is possible the BRIEF did not fully capture enough culturally relevant examples of cognitive flexibility. Examples include playing AI/AN games or children’s dual-language fluency, which may require EF skills such as cognitive flexibility and shifting to a greater extent [45,104]. Regardless, it is likely children utilized some aspects of cognitive flexibility to accomplish tasks associated with working memory and inhibition on the BRIEF. Future research should work with tribal communities to investigate examples of culturally relevant manifestations of EF to develop more accurate measures.

### 4.3. Disconnecting Forces and Urban AI Children’s EF

Community disconnectedness, as measured by parent experiences of discrimination, was negatively related to parent-rated child working memory, inhibition, and cognitive flexibility. Discrimination, prejudice, and stereotypes are widely linked with negative health outcomes for many communities, including impacts on physical and mental health, substance use, and the ability to find and maintain work [67,72]. Discrimination may be adding stress to the AI parents’ lives, and increased stressors are related to more negative biases, negatively influencing the lens through which parents view their children’s EF. This is further evidenced by the fact that discrimination dropped out as a significant predictor when children rated their own EF. It is likely that AI parents are working hard to shield their children from the effects of racism and discrimination in their communities. In contrast, neither family disconnectedness (stressful life events) nor environmental disconnectedness (neighborhood risk) were related to parent ratings of child EF, although neighborhood risk was associated with lower child self-rated EF. It may be that children are affected in different ways than parents by unsafe neighborhoods, especially considering that unsupervised children in the neighborhoods was reported as a top concern for AI parents in the current study. The added stress of these features of the environment may differentially influence how parents and children perceive their EF [47,49]. It is important to emphasize that findings for child self-reported EF were underpowered and should be interpreted with caution until future research is able to provide more insight. However, it does appear that discrepancies in the relation between disconnecting forces and parent versus child-rated EF may be related to which aspects of the environment cause more stress for the rater. Last, although family income was controlled for in all models, it is noteworthy that income was not related to parent or child-rated EF. This finding contradicts a wide body of literature on the negative effects of low socioeconomic status (SES) on child EF, even with diverse and low-income families [11,105,106]. The lack of association between low-income status and urban AI child EF echoes other emerging studies with AI populations [30,45,58]. These consistent null findings further emphasize the need to redefine and reconceptualize how the field measures “risk” for AI families.

### 4.4. Limitations and Future Directions

The present study is not without limitations. First, although small sample sizes are common in research with Indigenous communities, the small sample size may have impacted statistical power, particularly for the analyses utilizing the subsample of child self-report data. Second, the results may not generalize to other tribal communities. However, the present study adds significantly to the sparse data on risk and protective factors for urban AI children and offers preliminary evidence to support connecting and disconnecting forces as relevant for AI children’s executive function development. Third, while we were able to compare child self-reported EF with parent-reported EF, we did not have access to child self-reports of connecting and disconnecting forces. Nonetheless, the present study provides important information on these features as rated by the parent, which likely encompass risk and protective factors that impact the entire family. However, future research should explore age-appropriate ways to measure risk and protective factors with young children. Fourth, our analyses were not longitudinal and cannot demonstrate a causal relation between connecting and disconnecting forces and children’s EF. Power also limited our ability to test for moderation or mediation of connecting and disconnecting forces for children’s EF. Adding complexity is the possibility of bidirectional effects. For example, future research may wish to test whether cultural connectedness moderates the negative association between environment risks such as discrimination and children’s EF, but it is equally valid to test whether environment risks are moderating the positive association between cultural connectedness and children’s EF. Last, the present study was limited by the availability of culturally sensitive measures of both risk and protective factors, as evidenced by floor effects and lack of variability with some of the measures of disconnecting forces. While the present study chose to utilize subjective measures, which are shown to be better predictors of health outcomes, these measures were not without challenges. Some items used to measure connecting and disconnecting forces, as well as EF, may not have been culturally appropriate. For example, one item in the BRIEF described “getting too silly” as an indicator of poor EF. However, for AI families, this is not an indicator of poor behavioral or emotional regulation on the part of the child. Future research should utilize both qualitative and quantitative methods, centered in Indigenous theory, to reconceptualize and redesign measures of executive function for AI children.

## 5. Conclusions and Implications

Few studies have examined environmental risk factors alongside other risk and protective factors for urban American Indian (AI) communities or investigated their association with children’s executive function (EF). This exploratory study applied an Indigenous connectedness lens to examine risk and protective factors in urban AI children’s environments. Parents reported on connecting forces (knowledge of tribal history and engagement with cultural beliefs and traditional practices) and disconnecting forces (parent stressful life events, discrimination, and neighborhood risks) in their environment. Connecting forces were associated with higher EF for both parent-reported and child self-reported assessments, while disconnecting forces (discrimination and neighborhood risk) were related to lower child EF. Findings highlight the protective role of cultural connectedness for urban AI children’s cognitive development, even in the presence of disconnecting forces such as discrimination. The present study lays important groundwork for future research to develop culturally relevant measures of environmental risk for Indigenous families. Additionally, implications for policy and practice are profound. For example, policymakers, and practitioners could collaborate with Indigenous leaders to combat disconnecting forces such as discrimination by updating public school curricula to accurately reflect modern-day strengths and challenges for tribal communities in the U.S. Nearly 90% of all U.S. public school history textbooks teach AI/AN history using a pre-1900 context [107], which fosters harmful stereotypes and discrimination toward all AI/AN youth, particularly urban AI/AN youth attending public school. Policymakers and practitioners can also boost connecting forces for AI/AN youth by funding and promoting the development of urban cultural centers and tribal centers that provide a blend of cultural, spiritual, health, and education services for urban AI/AN youth and their families.

## Figures and Tables

**Table 1:** Correlations: demographics, connecting and disconnecting forces, and child EF.

	1	2	3	4	5	6	7	8	9	10	11	12	13	14
1. Child age	-													
2. Child sex	0.01	-												
3. Family income	−0.01	−0.13	-											
4. Neighbor. risks	0.01	0.16	0.06	-										
5. Stress. life events	0.12	0.12	−0.11	0.39 ***	-									
6. Tribal history	0.05	0.07	0.01	0.08	−0.01	-								
7. Discrimination	−0.15	−0.06	0.13	0.32 ***	0.15	0.15	-							
8. Culture. practices	−0.15	0.07	0.17	0.08	−0.12	0.38 ***	0.28 **	-						
9. P. Working mem.	−0.01	−0.10	−0.08	−0.13	−0.09	0.03	−0.20 **	0.18 *	-					
10. P. cog. flex.	0.07	0.03	−0.06	−0.01	−0.08	0.03	−0.23 *	0.10	0.58 ***	-				
11. P. inhibition	0.13	0.06	−0.13	0.02	0.01	0.20 *	−0.15	0.13	0.65 ***	0.58 ***	-			
12. C. working mem.	−0.13	0.04	−0.22	−0.15	−0.08	0.03	−0.09	0.04	0.07	−0.13	0.02	-		
13. C. cog. flex.	0.08	0.10	−0.17	−0.31 *	−0.13	0.24	−0.03	0.02	0.02	−0.22	0.12	0.72 ***	-	
14. C. inhibition	−0.17	0.02	−0.02	−0.31 *	−0.27	−0.01	0.10	0.40 **	0.23	0.12	0.14	0.74 ***	0.49 ***	-

Note. Pairwise spearman correlation coefficients presented. Average *n* = 102. Neighbor. risks = neighborhood risks. Stress. life events = stressful life events. Culture. practices = cultural beliefs and practices. Tribal history = knowledge and impact of tribal history. P. = parent-rated, C. = child-rated. Cog. flex. = cognitive flexibility. * *p* < 0.05, ** *p* < 0.01, and *** *p* < 0.001.

**Table 2:** Connecting and disconnecting forces and parent-reported EF (full sample).

	Working Memory		Inhibition		Cog. Flexibility	
	B (SE)	*p*	B (SE)	*p*	B (SE)	*p*
Neighborhood risk	0.001 (0.09)	0.99	0.09 (0.09)	0.34	0.11 (0.09)	0.25
Discrimination	−0.22 (0.10)	0.02	−0.23 (0.09)	0.01	−0.33 (0.09)	0.001
Stressful life events	−0.02 (0.09)	0.79	−0.06 (0.09)	0.55	−0.14 (0.09)	0.13
Cultural beliefs/practices	0.23 (0.11)	0.02	0.08 (0.10)	0.45	0.17 (0.09)	0.08
Tribal history	0.02 (0.10)	0.84	0.26 (0.10)	0.005	0.07 (0.09)	0.43

Note. *n* = 156. Standardized coefficients presented. All models control for income and child age. Cog. flexibility = cognitive flexibility.

**Table 3:** Connecting and disconnecting forces, and parent and child self-reported EF (ages 11–14).

	Parent-Rated EF						Child-Rated EF					
	Working Mem.		Inhibition		Cog. Flexibility		Working Mem.		Inhibition		Cog. Flexibility	
	β (SE)	*p*	β (SE)	*p*	β (SE)	*p*	β (SE)	*p*	β (SE)	*p*	β (SE)	*p*
Neighborhood risk	−0.12 (0.13)	0.33	−0.03 (0.13)	0.79	−0.01 (0.13)	0.91	−0.18 (0.17)	0.31	−0.22 (0.15)	0.14	−0.39 (0.15)	0.01
Discrimination	−0.26 (0.11)	0.02	−0.20 (0.11)	0.09	−0.33 (0.11)	0.003	0.04 (0.15)	0.81	0.19 (0.13)	0.14	0.11 (0.14)	0.41
Stressful life events	−0.05 (0.13)	0.72	−0.16 (0.13)	0.20	−0.21 (0.13)	0.11	−0.03 (0.19)	0.87	−0.01 (0.17)	0.97	0.05 (0.18)	0.78
Cultural beliefs/practices	0.31 (0.12)	0.01	0.19 (0.12)	0.13	0.07 (0.12)	0.57	0.16 (0.17)	0.33	0.45 (0.13)	0.001	−0.03 (0.15)	0.82
Tribal history	0.01 (0.12)	0.93	0.12 (0.12)	0.30	0.06 (0.11)	0.61	0.01 (0.16)	0.97	−0.08 (0.13)	0.55	0.21 (0.14)	0.12

Note. Parent-rated models *n* = 71, child-rated models *n* = 79. Standardized coefficients presented. All models control for income. Working mem. = working memory. Cog. flexibility = cognitive flexibility.

## Data Availability

The data and the materials necessary to reproduce the analyses and to attempt to replicate the findings are not publicly accessible due to ethical considerations and to protect the privacy, confidentiality, and anonymity of the vulnerable populations involved in this study. The statistical code is available upon request from the first author.

## Appendix A

**Table A1:** Disconnecting forces (environmental, community, and family).

Neighborhood Risks (Environmental)	*n*	*M* (SD)	Min	Max	Risk Present (%)
High unemployment	137	1.60 (0.72)	1.0	3.0	46.72
Not enough good housing	139	1.42 (0.61)	1.0	3.0	35.25
Rundown houses/abandoned cars	141	1.27 (0.48)	1.0	3.0	25.53
Police are not available	140	1.19 (0.45)	1.0	3.0	17.14
Car accidents	139	1.47 (0.65)	1.0	3.0	38.85
Gangs	141	1.23 (0.50)	1.0	3.0	19.86
Unsupervised children	140	1.54 (0.64)	1.0	3.0	45.71
Teenage pregnancy	139	1.19 (0.43)	1.0	3.0	17.27
Domestic Violence	140	1.39 (0.56)	1.0	3.0	35.00
Police and people do not get along	139	1.19 (0.40)	1.0	3.0	19.42
Broken homes/family breakups	138	1.31 (0.54)	1.0	3.0	27.54
Physical violence/abuse/neglect	138	1.28 (0.52)	1.0	3.0	23.91
Burglaries/theft	142	1.43 (0.56)	1.0	3.0	39.44
Alcohol abuse	140	1.39 (0.60)	1.0	3.0	32.14
Drug dealing	139	1.29 (0.48)	1.0	3.0	22.30
Total neighborhood risks	139	4.43 (4.65)	0.0	15.0	
Discrimination (Community)	*n*	*M* (SD)	Min	Max	Risk Present (%)
Problems in stores/restaurants	127	0.24 (0.47)	0.0	2.0	30.71
Cannot find work	125	0.34 (0.54)	0.0	2.0	20.00
Problems with police	127	0.22 (0.45)	0.0	2.0	22.83
Prejudice from White people	127	0.52 (0.59)	0.0	2.0	47.24
Total discrimination experiences	125	1.21 (1.40)	0.0	4.0	
Stressful Life Events (Family)	*n*	*M* (SD)	Min	Max	Risk Present (%)
Threatened by gang or violence	136	0.12 (0.32)	0.0	1.0	11.76
Someone important attempted suicide	134	0.19 (0.39)	0.0	1.0	18.66
Someone important committed suicide	136	0.07 (0.26)	0.0	1.0	7.35
Car accident/other serious accident	135	0.13 (0.33)	0.0	1.0	12.59
Family violence	136	0.39 (0.49)	0.0	1.0	38.97
Physical abuse (partner)	135	0.19 (0.39)	0.0	1.0	18.52
Physical abuse (non-partner)	136	0.20 (0.40)	0.0	1.0	19.85
Attacked outside the home	136	0.18 (0.39)	0.0	1.0	18.38
Direct combat experience	136	0.05 (0.22)	0.0	1.0	5.15
Total stressful life events	135	1.52 (1.79)	0.0	7.0	

**Table A2:** Connecting forces (spiritual, intergenerational).

Cultural Beliefs/Traditional Practices (Spiritual)	*n*	*M* (SD)	Min	Max	% Agree	% Strongly Agree
Being a part of my tribe important to me	128	4.22 (1.03)	1.0	5.0	29.69	51.56
My life is affected by being American Indian	128	3.23 (1.28)	1.0	5.0	30.47	18.75
I have a lot of pride in my tribe	128	4.16 (1.05)	1.0	5.0	29.69	49.22
I speak/learning to speak tribal language	128	3.69 (1.25)	1.0	5.0	35.16	30.47
I follow tribal religious/spiritual traditions	128	3.43 (1.33)	1.0	5.0	26.56	26.56
I listen/sing/dance to tribal music	128	3.23 (1.42)	1.0	5.0	20.31	25.78
I have a strong sense of belonging to tribe	128	3.75 (1.20)	1.0	5.0	28.91	34.38
I learn about my tribe or culture	128	3.89 (1.10)	1.0	5.0	34.38	35.16
I feel good about my tribal background	127	4.29 (0.97)	1.0	5.0	29.13	54.33
Average cultural beliefs/traditional practices	128	3.71 (0.79)	0.0	5.0		
Knowledge of Tribal History (Intergenerational)	*n*	*M* (SD)	Min	Max	% “Some”	% “A lot”
How much do you think about tribal history?	133	2.10 (0.60)	1.0	3.0	63.16	23.31
How much do you know about tribal history?	134	1.99 (0.59)	1.0	3.0	65.67	16.42
How big an impact tribal history on personal life?	134	2.02 (0.72)	1.0	3.0	48.51	23.13
How big an impact tribal history on community?	134	2.05 (0.64)	1.0	2.0	58.96	26.87
Average knowledge/impact of tribal history	133	1.99 (0.61)	1.0	3.0		

**Table A3:** Executive function (parent and child-rated BRIEF subscales).

	Parent Ratings (Full Sample)				Child Ratings (11–14)				Parent Ratings (11–14)			
	*n*	*M* (SD)	Min	Max	*n*	*M* (SD)	Min	Max	*n*	*M* (SD)	Min	Max
BRIEF working mem.	138	2.41 (0.49)	1.00	3.00	56	2.38 (0.41)	1.42	3.00	71	2.41 (0.45)	1.00	3.00
BRIEF inhibition	138	2.57 (0.42)	1.00	3.00	56	2.57 (0.35)	1.50	3.00	71	2.63 (0.40)	1.00	3.00
BRIEF cog. flexibility	138	2.56 (0.41)	1.14	3.00	57	2.44 (0.34)	1.60	3.00	71	2.60 (0.43)	1.14	3.00

Note: Working mem. = working memory. Cog. flexibility = cognitive flexibility.
